# Skull Base Osteomyelitis With Bilateral Acute Otitis Media

**DOI:** 10.7759/cureus.24144

**Published:** 2022-04-14

**Authors:** Noora Althawadi, Amr Elsayed M Hussein, Omar Sabra

**Affiliations:** 1 Ear, Nose, Throat (ENT), Northwick Park Hospital, London, GBR; 2 Radiology, King Hamad University Hospital, Muharraq, BHR; 3 Otolaryngology - Head and Neck Surgery, King Hamad University Hospital, Muharraq, BHR

**Keywords:** necrotizing external otitis, pet scans, bilateral hearing loss, skull base osteomyelitis, chronic otitis media

## Abstract

Skull base osteomyelitis is an inflammatory process that usually occurs secondary to necrotizing otitis externa or chronic mastoid infections. The involvement of the external auditory canal is typical of this condition and aids in its diagnosis. The treatment of skull base osteomyelitis is often complex and involves long-term intravenous antibiotics.

Skull base osteomyelitis originating from the middle ear is a rare entity. We report a case of skull base osteomyelitis originating from the bilateral otitis media.

## Introduction

Lateral skull base osteomyelitis (SBO), otherwise known as necrotizing otitis externa (NOE), is a severe invasive infection of the external auditory canal, temporomandibular joint, and skull base, which occurs by spread from the external ear canal through the fissures of Santorini and the osseocartilaginous junction. Diagnosis of NOE is based on history, physical examination, inflammatory markers, and radiological studies. Elevated C-reactive protein (CRP) and erythrocyte sedimentation rate (ESR) are classically seen in patients with NOE. CT scanning allows for the identification of bony erosion. Technetium Tc 99m bone scans also allow for diagnosing bony involvement. Although the Gallium scan is the gold standard for following up patients with necrotizing otitis externa, FDG-PET/CT scan has shown to be a reliable alternative when gallium is not available [1-2].

Due to its aggressive nature and associated high morbidity and mortality, prolonged treatment with broad-spectrum antibiotics and possible surgical requirement is the mainstay treatment option for skull base osteomyelitis [3]. In addition, improving the oxygen perfusion of tissues is thought to strengthen host defenses and lead to oxidative damage of pathogens. This can be done by using hyperbaric therapy as an adjunct to the treatment of SBO [4].

SBO originating from the otitis media is a rare entity. We report a case of SBO occurring in a gentleman with bilateral otitis media, in the absence of external ear disease.

## Case presentation

A 74-year-old diabetic gentleman with chronic kidney disease presented to the Ear, Nose, and Throat Clinic with a history of prolonged ear pain and hearing loss associated with ear drainage bilaterally. On examination, he was found to have bilateral ear discharge. The patient was started on oral co-amoxiclav and topical ear drops for suspicion of perforated acute otitis media in view of the quality of drainage and the bilaterality of the disease. Initial ear canal culture from the drainage was positive for *Klebsiella Aerogenes* resistant to co-amoxiclav.

Upon one-week follow-up, the patient continued to complain of ear pain and headaches. The tympanic membranes were bilaterally intact, however, both were bulging with granulation tissue covering the tympanic membrane. This was seen more on the right side with right ear discharge. The left ear canal was erythematous and the tympanic membrane was dull. A myringotomy was done bilaterally. Serous fluid was drained from the left ear, but only hyperplastic mucosa and a thickened drum were encountered in the right ear, which was previously draining. Both ears were cultured during the procedure, including the granulation tissue, but these results came back negative.

A decision was made to commence the patient on intravenous ceftazidime and topical ciprofloxacin ear drops to cover *Klebsiella Arogene*s, as it was the only pathogen that was grown in previous culture results. Initial blood tests showed a high erythrocyte sedimentation rate (ESR) and C-reactive protein (CRP); ESR 56 (normal lab range 0-20) and CRP 44.9 (normal lab range 0-3). The prolonged history of symptoms and failure to respond to topical and systemic treatment lead to the suspicion of skull base osteomyelitis.

**Figure 1:**
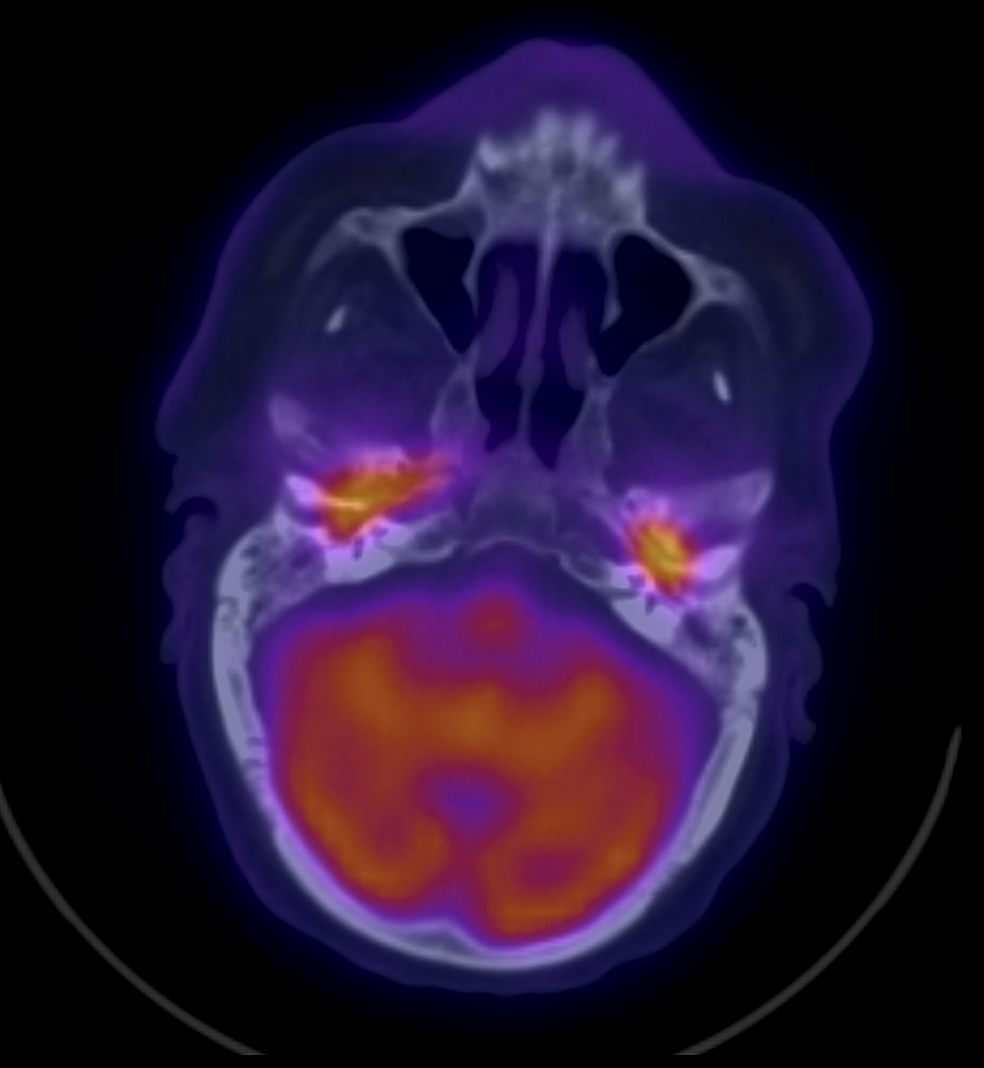
Fused transaxial F-18 FDG-PET/CT showing bilateral hypermetabolic involvement of both middle ears without direct extension from one side to the other through the skull-base structures FDG-PET: fluorodeoxyglucose-positron emission tomography

A non-contrast CT scan was ordered in view of the patient’s chronic kidney disease, and it reported the following: Bilaterally thickened tympanic membranes with worsening of middle ear and mastoid opacification with evidence of erosion into the temporomandibular joint (TMJ), more advanced on the right side. This was followed by an F-18 fluorodeoxyglucose (FDG)-positron emission tomography/computed tomography (F-18 FDG-PET/CT) scan, which reported the following: intense FDG activity is seen opposite the CT-detected soft tissue densities occupying the middle ears as well the tympanic part of temporal bones and mastoid antrum on both sides and anterior aspect of right petrous part of the temporal bone (Figure 1).

The intravenous antibiotic was changed to a broader spectrum (piperacillin-tazobactam) to cover the classical necrotizing otitis externa pathogens. The patient was also started on oral ciprofloxacin along with topical ear drops, regular aural toileting, and hyperbaric therapy. The patient showed improvement in his overall clinical status. He was complaining of reduced pain and improved hearing. On examination, there was granulation tissue over the tympanic membranes on both sides but the resolution of the middle ear effusion. His CRP and ESR showed a downward trend at first and then stabilized at a relatively high level.

After completing two months of treatment, the treatment was stopped for one week during which the clinical picture worsened, with re-drainage starting from the ear canal. On follow-up FDG PET/CT scan, there was a significant worsening in FDG activity in both middle ears with an extension of the activity into the parapharyngeal spaces and occipital skull base area. Treatment was resumed in the outpatient setting. Grommets were inserted bilaterally under local anesthesia. Serous fluid was drained from both ears. After approximately one month of intravenous outpatient treatment, no clinical nor laboratory improvement could be achieved on antipseudomonal antibiotics covering the previously seen pathogen.

**Figure 2:**
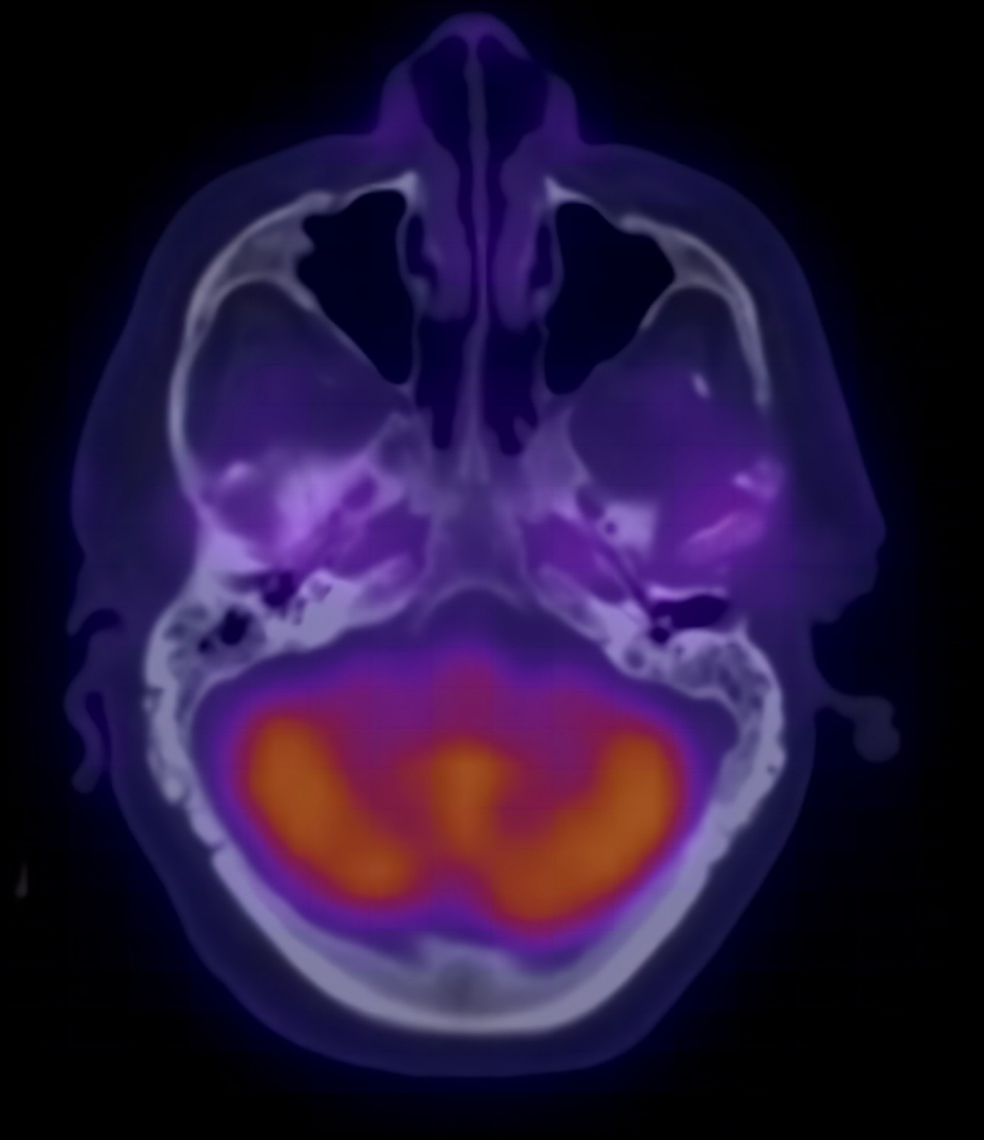
Follow-up fused transaxial F-18 FDG-PET/CT after six months of antimicrobial treatment showing resolution of abnormal FDG uptake of both middle ears FDG-PET: fluorodeoxyglucose-positron emission tomography

The patient was readmitted and middle exploration was done with tissue culture taken from both the scutum and middle ear mucosa on both sides. Both cultures showed *Aspergillus Flavus* and *Candida Parapsilosis*. The patient was then started on intravenous voriconazole with significant clinical and laboratory improvement in both infectious markers, CRP and ESR, until they normalized. A repeat FDG-PET/CT scan also showed resolution of inflammation bilaterally (Figure 2).

## Discussion

Skull base osteomyelitis (SBO) is an inflammatory process that may involve the frontal, sphenoidal, temporal, and occipital bones, caused by an infection or direct head trauma (injuries and iatrogenic trauma) [5]. Temporal bone involvement usually occurs secondary to necrotizing otitis externa (NOE) or chronic mastoid infections [6]. The major risk factor for the development of NOE is well-known to be uncontrolled diabetes mellitus, typically in an elderly patient. Both risk factors were present in our patient.

*Pseudomonas aeruginosa* is the causative pathogen in at least 95% of NOE cases. Patients who have NOE commonly complain of severe otalgia that is not comparable to the clinical findings, and otorrhea that does not respond to first-line topical treatment given for otitis externa [1]. Even though the patient had prolonged ear pain that did not respond to initial therapy, his symptoms were not always associated with otorrhea and early in the disease, he presented with a bulging tympanic membrane and ear effusion. This rules out the presence of external ear disease. His ear swabs also did not grow *Pseudomonas Aeroginosa*, an expected culprit in SBO originating from otitis externa.

Other key points mentioned in the case report above point out the underlying otitis media leading to SBO. The fact that the patient had concurrent bilateral disease as evident by his symptoms and the early FDG-PET/CT scans done, without a direct extension from one side to another through the skull base, further suggests the primary infection is bilateral otitis media, as opposed to otitis externa, which is typically unilateral. A case of bilateral NOE has been reported by T. Leahy and C. Sader [7], however, the disease initially affected the right side. After resolution, the patient developed symptoms on the left side.

Furthermore, patients with NOE are commonly found to have granulation tissue in the external auditory canal, especially at the osseocartilaginous junction. The site of granulation tissue found in our patient is atypical to NOE, as there was an absence of granulation tissue in the external auditory canal, and it was present over the tympanic membrane only.

SBO originating from otitis media is rarely reported in the literature. Very few case reports have been published discussing this pathology. A case report published by Merchant and Vernick in 1992 discusses the development of SBO in a gentleman who was treated for otitis media with ventilation tubes, by allowing the organism to invade the external auditory meatus [8]. Another case of skull base originating from otitis media has been reported by Low and Lhu in 2018. Similarly, the organism gained access to the external auditory meatus through a perforation in the tympanic membrane [9]. Our case is different, as there was no initial communication between the middle ear and external ear, especially on the left side. In addition, the presence of bony activity on FDG-PET/CT scan bilaterally around the middle ears, suggests a possible direct extension to the skull base bone from the middle ear without going through the classical outer ear intermediate.

## Conclusions

Skull base osteomyelitis resulting from otitis media is an emerging entity that needs to be further studied in detail. The involvement of atypical pathogens makes it a more difficult disease to treat compared to necrotizing otitis externa. F-18 FDG-PET/CT should be considered to diagnose questionable necrotizing otitis externa, evaluate the disease extent, and assess response to antimicrobial therapy.
